# Development and validation of nomogram for predicting neurogenic pulmonary edema in hypertensive intracerebral hemorrhage

**DOI:** 10.3389/fcvm.2026.1726478

**Published:** 2026-02-03

**Authors:** Yajuan Xu, Yinxian Shi

**Affiliations:** 1 Department of ICU, Suzhou Hospital of Integrated Traditional Chinese and Western Medicine, Suzhou, Jiangsu, China; 2 Department of Nursing, Suzhou Hospital of Integrated Traditional Chinese and Western Medicine, Suzhou, Jiangsu, China

**Keywords:** hypertensive, intracerebral hemorrhage, neurogenic pulmonary edema, nomogram, risk

## Abstract

**Objective:**

The pathophysiological mechanism of neurogenic pulmonary edema (NPE) is still unclear, and the condition is severe with a high mortality rate. Identifying the risk factors for NPE is of great significance. Therefore, this study aims to explore the risk factors of hypertensive intracerebral hemorrhage (HICH) complicated with NPE and construct a risk prediction model based on this.

**Methods:**

We retrospectively collected baseline admission data from 274 patients with hypertensive intracerebral hemorrhage (HICH) admitted to Suzhou Hospital of Integrated Traditional Chinese and Western Medicine from March 2023 to March 2025. All candidate predictors were obtained from the first clinical assessment within 6 h after admission (first available values), including vital signs, GCS score, arterial blood gas parameters (PaO2/FiO2 and lactate), BNP, and hematoma volume measured on the initial CT scan. Patients were classified into NPE and non-NPE groups according to the occurrence of new-onset NPE during hospitalization. Apply the Least Absolute Shrinkage and Selection Operator (LASSO) method and multivariate logistic regression to determine independent risk factors. These risk factors are used to construct a nomogram for predicting the risk of NPE occurrence. Additionally, 152 patients with HICH from January 2022 to March 2023 were collected as an internal temporal validation cohort. Compared through consistency index (C-index), calibration curve, receiver operating characteristic (ROC) curve, and DCA.

**Results:**

The cerebral hemorrhage volume, heart rate, GCS score, arterial partial pressure of oxygen/fraction of inspired oxygen (PaO2/FiO2), Lactic (LAC) and B-type natriuretic peptide (BNP) are independent influencing factors of HICH complicated with NPE (*P* < 0.05). Establish and validate a risk prediction model for HICH concurrent NPE based on the above six risk factors. The C-index of the training cohort and validation cohort are 0.980 (95% CI: 0.966–0.994) and 0.976 (95% CI: 0.952–1.000), respectively. The calibration curve of the nomogram shows good consistency with the ideal curve. The ROC curves showed that the AUC of NPE risk in the training cohort and validation cohort patients were 0.985 (95% CI: 0.973–0.997) and 0.975 (0.955–0.996), respectively; The DCA shows that HICH patients have a higher net benefit in predicting the risk of NPE based on this model.

**Conclusions:**

The nomogram has good predictive performance and applicability for predicting NPE in HICH. This can be used to screen for the risk of NPE occurrence in this population.

## Introduction

Hypertensive intracerebral hemorrhage (HICH) is one of the severe types of acute stroke (1, 2). HICH is a dangerous condition, difficult to treat, and has a high disability and mortality rate (2). Reports shows that among all patients with cerebral hemorrhage, hypertension accounts for over 70% (1–3). The incidence rate of HICH increases exponentially with age (2–4). China is a country with high blood pressure, and the occurrence of HICH brings a heavy burden to families and society (4, 5). Previous research shows that in 2020, HICH patients accounted for 14.2% of the stroke population in China, with an average age of onset of 66 years, and more males than females (3–5). The clinical prognosis of HICH is poor, often accompanied by various complications such as neurological damage, cerebral edema, pulmonary edema, gastrointestinal bleeding, etc. (5, 6). Among them, neurogenic pulmonary edema (NPE) is an acute respiratory distress syndrome (ARDS) characterized by acute attacks and rapid accumulation of interstitial fluid in the lungs (6, 7). The reports shows that the incidence of NPE ranges from 2% to 42.9% (6–9). The analysis of factors related to NPE after cerebral hemorrhage showed that the incidence of NPE was 18% (9). NPE typically occurs acutely within 4 h or has a delayed onset within 12–72 h (8, 9). The incidence in critically ill patients (GCS score ≤ 8) can reach 60%–100% (5, 10).

At present, the pathogenesis of NPE is still unclear. Some scholars believe that significant sympathetic nervous system excitation and release of vasoactive substances are involved in the process of NPE (2, 8, 9). Due to the fact that NPE occurs within a few hours of HICH, with a rapid onset and progression, the mortality rate is extremely high (10). Critically ill NPE patients not only suffer from respiratory failure, but also exacerbate neurological damage and functional failure of other organs due to reduced effective circulating blood volume (8–10). Therefore, it is of great significance to clarify the risk factors for HICH complicated with NPE. This can identify high-risk groups for NPE as early as possible and minimize the incidence of NPE to the greatest extent possible.

To our knowledge, there are relatively few reports on the risk factors for HICH concurrent NPE, especially the lack of nomograms used to predict HICH concurrent NPE. This study aims to develop a practical and reliable nomogram for predicting the occurrence of NPE by combining common clinical variables. We hope to help clinical workers identify high-risk populations of HICH complicated with NPE as early as possible.

## Materials and methods

### Patients

We retrospectively selected 274 patients with HICH admitted to Suzhou Hospital of Integrated Traditional Chinese and Western Medicine from March 2023 to March 2025 as the training cohort. In addition, we also collected 152 patients with HICH from January 2022 to March 2023 as an internal temporal validation cohort. The later period was used for model development because clinical workflows and key laboratory testing at admission were more standardized and electronically recorded, resulting in more complete baseline measurements and more consistent variable definitions. The earlier period was used for validation as a conservative assessment of model robustness under less standardized real-world conditions, which reduces the risk of overly optimistic validation driven by temporal improvements in data capture and clinical management.

Ethics approval: This study was approved by the Ethics Committee of Suzhou Hospital of Integrated Traditional Chinese and Western Medicine (Approval No. 2025030).

Inclusion criteria:

- Meets the relevant diagnostic criteria in the Chinese Multidisciplinary Diagnosis and Treatment Guidelines for Hypertensive Cerebral Hemorrhage (11);
- Age ≥ 18 years old;
- History of hypertension;
- Imaging shows typical bleeding sites such as thalamus, cerebellum, and basal ganglia;
- Complete clinical data were available;

Exclusion criteria:

- Patients with cerebral vascular abnormalities such as intracranial aneurysms, cerebral vascular malformations, and moyamoya disease;
- Patients with combined malignant tumors;
- Patients with coagulation dysfunction and hematological disorders;
- Patients with severe dysfunction of major organs, including the heart, kidneys, liver, or lungs;
- Patients with sudden cardiac arrest or undergoing cardiopulmonary resuscitation;
- Patients who met NPE diagnostic criteria at admission.

NPE diagnosis: NPE was diagnosed according to prespecified criteria (6). The primary endpoint was new-onset NPE during hospitalization after admission, and outcome adjudication was performed during the subsequent clinical course based on medical records, laboratory results, and imaging findings. The diagnostic criteria were as follows:

1) New-onset respiratory distress during the course of intracerebral hemorrhage, including dyspnea, tachypnea, cyanosis, tachycardia, and auscultatory findings such as rales or wheezing. 2) Respiratory rate ≥ 30 breaths/min and PaO2/FiO2 ≤ 200 mmHg. 3) Chest radiography suggestive of pulmonary edema, typically with bilateral diffuse alveolar infiltrates, with radiographic resolution within 48–72 h after onset. 4) Exclusion of other causes of pulmonary edema, including cardiogenic pulmonary edema, aspiration pneumonia, pneumonia, sepsis, negative-pressure pulmonary edema, post-obstructive pulmonary edema, ventilator-associated pneumonia, ventilator-induced lung injury, and transfusion-related lung injury.

Outcome adjudication workflow: To ensure reproducibility in retrospective review, NPE adjudication was operationalized using a prespecified chart-review checklist. Two trained reviewers independently assessed each suspected case according to the predefined criteria and the overall clinical course, and disagreements were resolved by consensus. Alternative causes of pulmonary edema were systematically evaluated using available documentation, including symptoms and physical findings, infection-related laboratory results and microbiological tests when available, chest imaging patterns and evolution, fluid balance records, renal function, transfusion records, and mechanical ventilation–related documentation. Cardiogenic pulmonary edema was excluded through a structured review of cardiac-related evidence, including documented history or clinical evidence of heart failure or significant structural heart disease, electrocardiography and cardiac biomarkers when available, and echocardiographic findings when performed, with attention to left ventricular systolic function and major valvular abnormalities. Cases with persistent pulmonary congestion together with objective evidence of cardiac dysfunction, or with evidence supporting pneumonia or aspiration, sepsis-related lung injury, volume overload or renal failure–related pulmonary edema, transfusion-related lung injury, ventilator-associated pneumonia, ventilator-induced lung injury, or other non-neurogenic etiologies were adjudicated as non-NPE.

### Data collection

Clinical data were extracted from the hospital information system and electronic medical records, including demographic characteristics, medical history, neurological status (GCS score), radiological variables (hematoma volume and location), vital signs (heart rate, systolic and diastolic blood pressure, and oxygen saturation), and laboratory variables (arterial blood gas parameters including PaO2/FiO2 and lactate, BNP, and other routine biomarkers). The primary endpoint was new-onset NPE during hospitalization. To minimize time-dependent bias, the index time was defined as hospital admission to the emergency department or ICU. All candidate predictors were obtained from the first clinical assessment within 6 h after admission using the first available values, rather than the worst values during hospitalization or values recorded at respiratory deterioration. Specifically, heart rate, blood pressure, and oxygen saturation were taken from the first recorded vital signs at ED or ICU admission; the GCS score from the initial neurological examination; PaO2/FiO2 and lactate from the first arterial blood gas analysis after admission; BNP from the first venous blood sample collected at admission; and hematoma volume was calculated based on the initial head CT scan.

### Statistical analysis

Data are entered into Microsoft Excel and analyzed using SPSS version 26.0 (IBM Corp, Armonk, NY, USA). Categorical variables were expressed as *n* (%), and between-group differences were assessed using the chi-square test; For example, gender, smoking, drinking, diabetes, hyperlipidemia, bleeding location, etc. Visual (histogram and probability plot) and analytical (Kolmogorov Smirnov/Shapiro Wilk test) methods are used to evaluate whether variables follow a normal distribution. Non normally distributed data are represented by median and interquartile range, and Mann Whitney U test is used for inter group comparison. Use the Least Absolute Shrinkage and Selection Operator (Lasso) regression to screen for independent prognostic factors. Then merge the predictor variables selected by Lasso regression into logistic regression to construct a nomogram. Based on the predictive model, the performance of the nomogram was evaluated in both the training cohort and validation cohort. The receiver operating characteristic curve (ROC) and calibration curve are used to evaluate the predictive performance of the model. The area under curve (AUC) of the ROC curve ranges from 0.50 to 1.00, and the closer the AUC approaches 1, the better the prediction performance. The calibration process checks whether the predicted risks are consistent with the observed risks. To evaluate the clinical utility of the model in predicting NPE occurrence, decision curve analysis (DCA) was used to assess its effectiveness. A two-sided *P* < 0.05 was considered statistically significant.

## Results

### Clinical data

**Figure 1:**
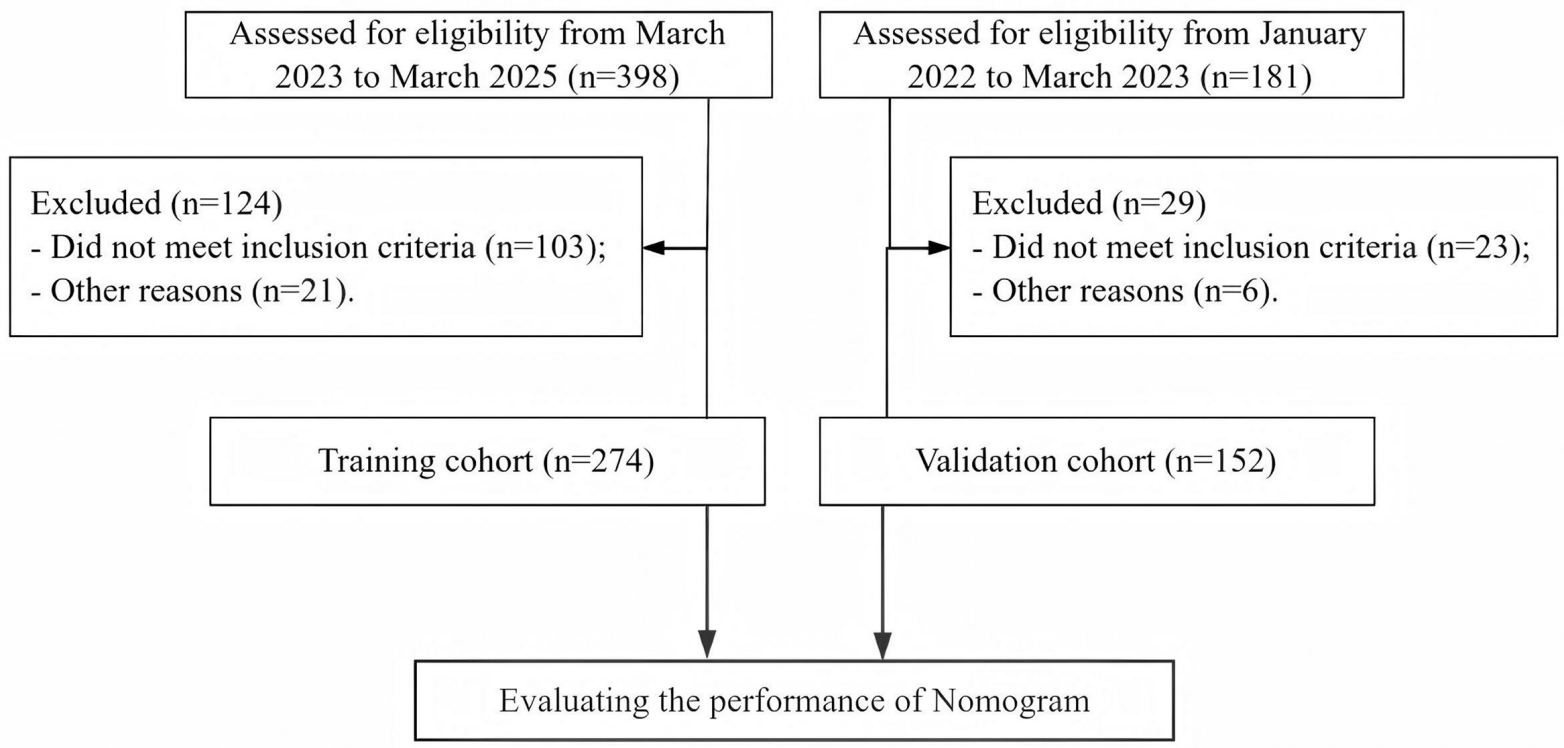
Patient screening process diagram.

We retrospectively selected clinical data of 274 patients with HICH admitted to our hospital from March 2023 to March 2025. In addition, we also collected 152 patients with HICH from January 2022 to March 2023 as an internal temporal validation cohort. The screening process is shown in Figure 1, and the clinical characteristics of the patients are shown in Table 1. The incidence of NPE in the training cohort and validation cohort was 19.3% (53/274) and 22.4% (34/152), respectively, with no statistically significant difference between the two cohorts (*χ* 2 = 0.551, *P* = 0.458).

**Table 1:** Basic information of the patient.

Variables	Training cohort (*n* = 274)	Validation cohort (*n* = 152)	Z/*χ^2^*	*P*
Age (years),	59 (55–65)	60 (54–65)	−0.297	0.766
Male (yes), *n* (%)	155 (56.6)	81 (53.3)	0.426	0.514
BMI (kg/m^2^)	23.6 (20.6–25.4)	22.45 (20.45–24.85)	−1.750	0.080
Smoke (yes), *n* (%)	142 (51.8)	84 (55.3)	0.464	0.496
Alcohol (yes), *n* (%)	110 (40.1)	55 (36.2)	0.647	0.421
Diabetes (yes), *n* (%)	80 (29.2)	50 (32.9)	0.630	0.427
Hyperlipidemia (yes), *n* (%)	57 (20.8)	43 (28.3)	3.050	0.081
Bleeding location (yes), *n* (%)			4.666	0.198
Basal ganglia	120 (43.8)	58 (38.2)		
Thalamus	70 (25.5)	32 (21.1)		
Brain lobes	46 (16.8)	36 (23.7)		
Brain stem	38 (13.9)	26 (17.1)		
Cerebral hemorrhage volume (mL)	34 (28–38)	35 (28.5–41)	−1.465	0.143
GCS score	9 (8–11)	9 (8–11)	−0.584	0.559
HR (beats/min)	94 (83–105)	90 (84–104.5)	−0.196	0.844
PaO2/FiO2 (mmHg)	265 (199–305)	256.5 (187–323.5)	−0.420	0.674
PCO2 (mmHg)	39.4 (36.4–42.3)	38.6 (35.95–42.1)	−1.050	0.294
HCO3- (mmol/L)	22.5 (20.4–24.4)	22.1 (20.8–23.65)	−1.244	0.214
LAC (mmol/L)	3.28 (2.76–3.6)	3.34 (2.86–3.66)	−0.417	0.677
BNP (pg/mL)	255 (198–287)	233 (156–321)	−1.614	0.107
hs-CRP (mg/L)	9.27 (8.27–12.09)	10.15 (8.81–12.18)	−1.097	0.273
NPE (yes), *n* (%)	53 (19.3)	34 (22.4)	0.551	0.458

BMI, body mass index; GCS, Glasgow Coma Scale; HR, heart rate; PaO2/FiO2, arterial partial pressure of oxygen/fraction of inspired oxygen; PCO2, arterial carbon dioxide; HCO3–, bicarbonate, LAC, Lactic; BNP, B-type natriuretic peptide; hs-CRP, high-sensitivity C-reactive protein.

### Predictive factors for NPE occurrence after HICH

**Figure 2:**
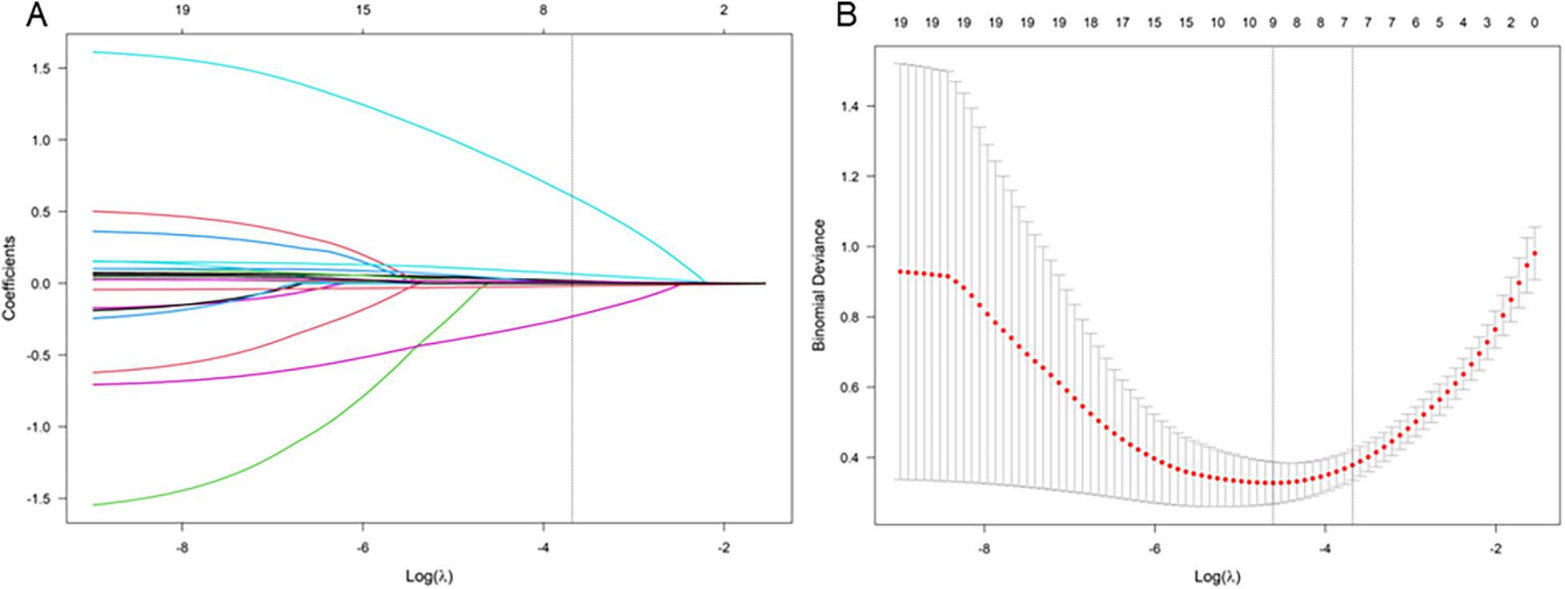
LASSO coefficient curve of NPE occurring after HICH. **(A)** Each curve in the graph represents the coefficient variation of each variable. The vertical axis represents the coefficient values, the lower horizontal axis represents log (*λ*), and the upper horizontal axis represents the number of non-zero coefficients in the model at this time. **(B)** 10 fold cross validation fitting, then select the model.

Firstly, we preliminarily selected the predictive factors for NPE occurrence through LASSO regression; The variables were centralized and normalized through 10-fold cross-validation (Figure 2). The selected predictive factors are cerebral hemorrhage volume, HR, GCS score, PaO2/FiO2, LAC, and BNP levels. Secondly, six predictive factors were included as independent risk variables, and a predictive model was constructed using multivariate logistic regression (Table 2); These six predictive factors are cerebral hemorrhage volume (OR: 1.204; 95% CI: 1.016∼1.426), heart rate (1.067; 1.005∼1.133), GCS score (0.445; 0.250∼0.792), PaO2/FiO2 (0.934; 0.901∼0.968), LAC (3.428; 1.204∼9.759), and BNP (1.026; 1.009∼1.044).

**Table 2:** Multivariate logistic regression analysis of predictive factors selected through LASSO regression program for model development.

Independent variables	*B*	OR (95% CI)	*P*
Cerebral hemorrhage volume	0.185	1.204 (1.016∼1.426)	0.032
HR	0.065	1.067 (1.005∼1.133)	0.034
GCS score	−0.809	0.445 (0.250∼0.792)	0.006
PaO2/FiO2	−0.068	0.934 (0.901∼0.968)	<0.001
LAC	1.232	3.428 (1.204∼9.759)	0.021
BNP	0.026	1.026 (1.009∼1.044)	0.003

HR, heart rate; GCS, Glasgow Coma Scale; PaO2/FiO2, arterial partial pressure of oxygen/fraction of inspired oxygen; LAC, Lactic; BNP, B-type natriuretic peptide.

### Nomogram of NPE occurrence after HICH

**Figure 3:**
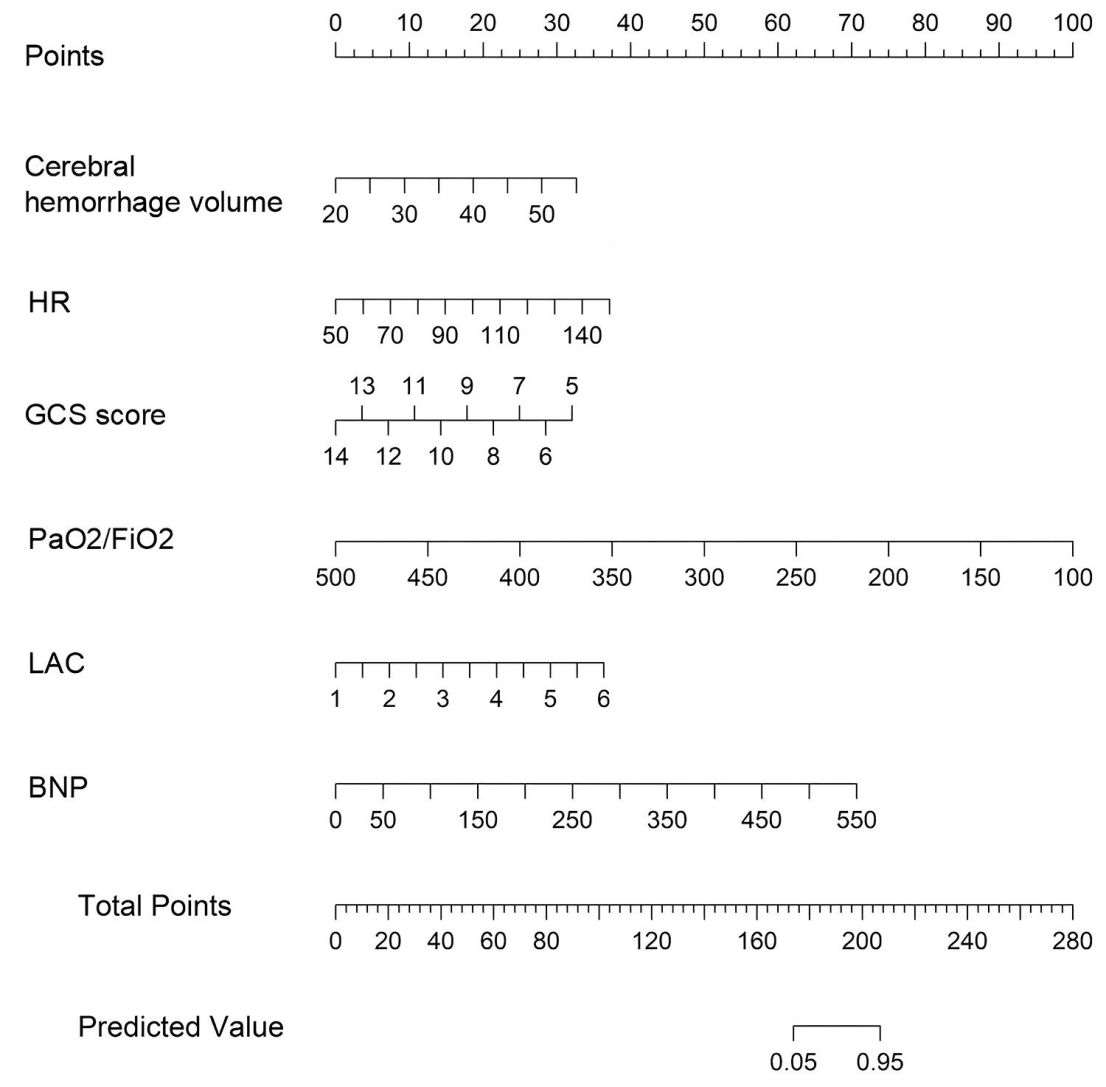
Nomogram for predicting NPE occurrence after HICH. Each level of the predictor variable represents a specific score. The total score is generated by summarizing the scores of each predictor variable. The total score corresponds to the probability of NPE.

Construct a nomogram for predicting NPE occurrence after HICH based on the six independent risk factors mentioned above (Figure 3). According to the score values corresponding to each predictive indicator in the nomogram, the sum of these score values is recorded as the total score, and the predicted probability corresponding to the total score is the risk of NPE in patients with HICH.

### Calibration and validation of nomogram

**Figure 4:**
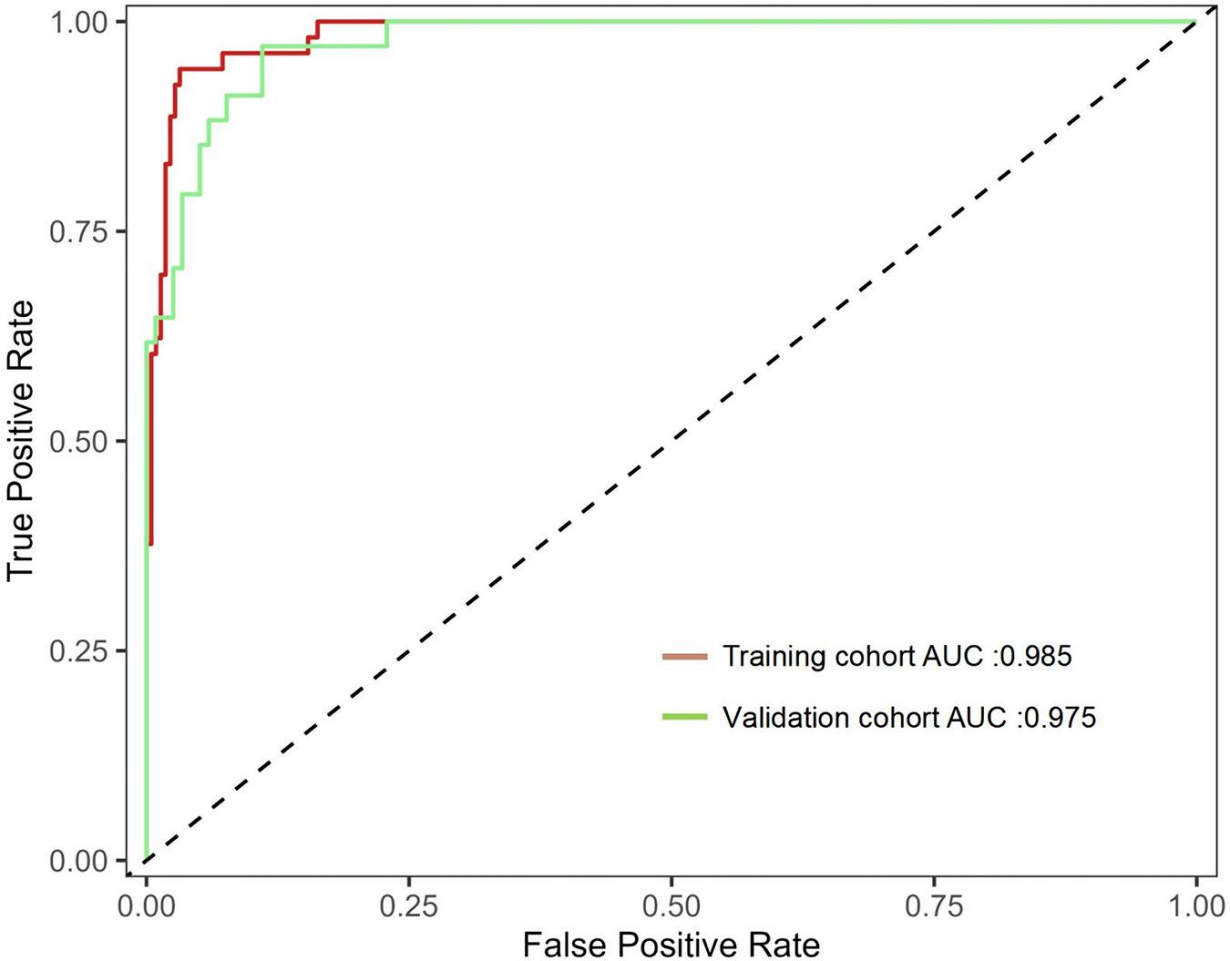
Shows the ROC curve and AUC of the prediction model. ROC, receiver operating characteristic; AUC, area under the curve.

**Figure 5:**
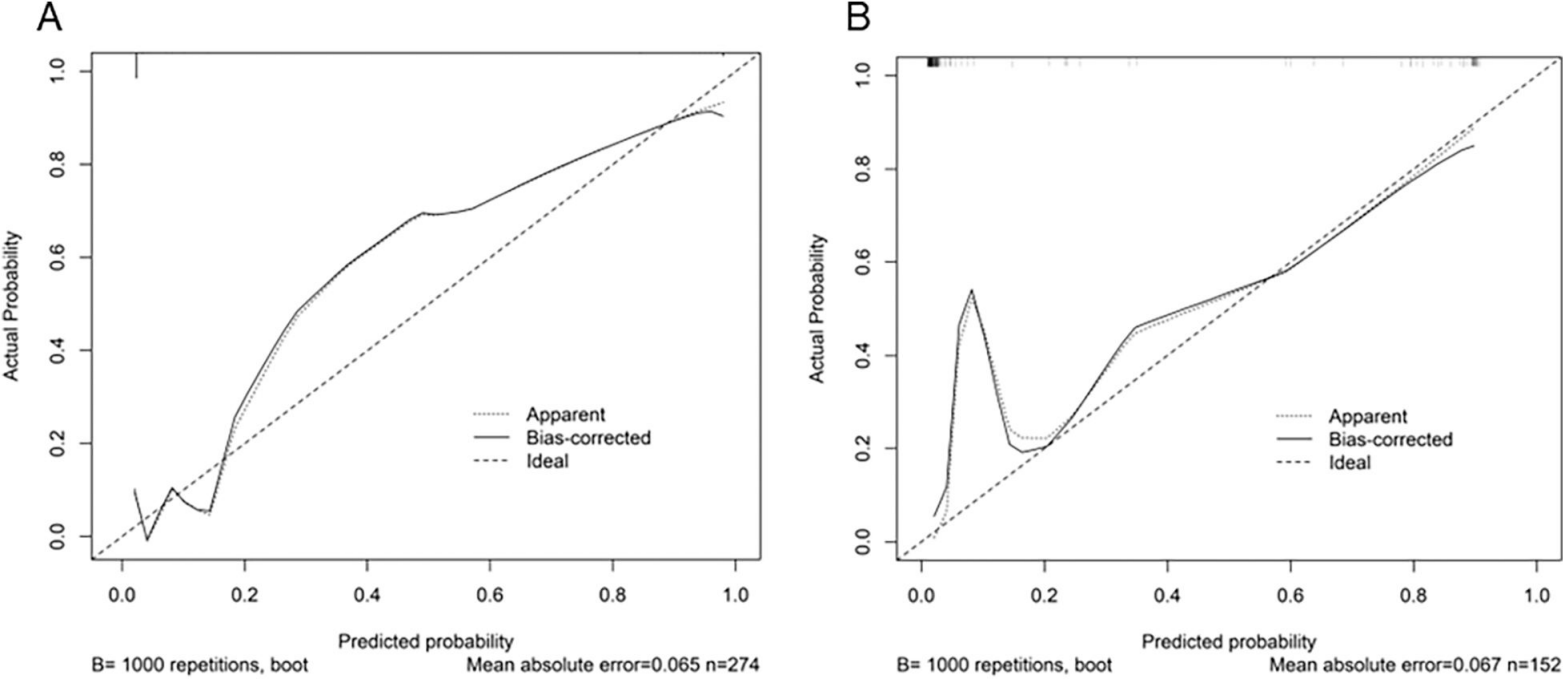
Calibration diagram of the prediction model. **(A)** Calibration chart of the training cohort. **(B)** Verify the calibration chart in the cohort. The *x*-axis represents the predicted probability of NPE. The *y*-axis represents the observed NPE. The diagonal dashed line represents the perfect prediction of the ideal model. The solid line represents the performance of the nomogram. It indicates that solid lines are closer to diagonal dashed lines for better prediction. From the figure, it can be seen that the prediction model has good predictive ability.

**Figure 6:**
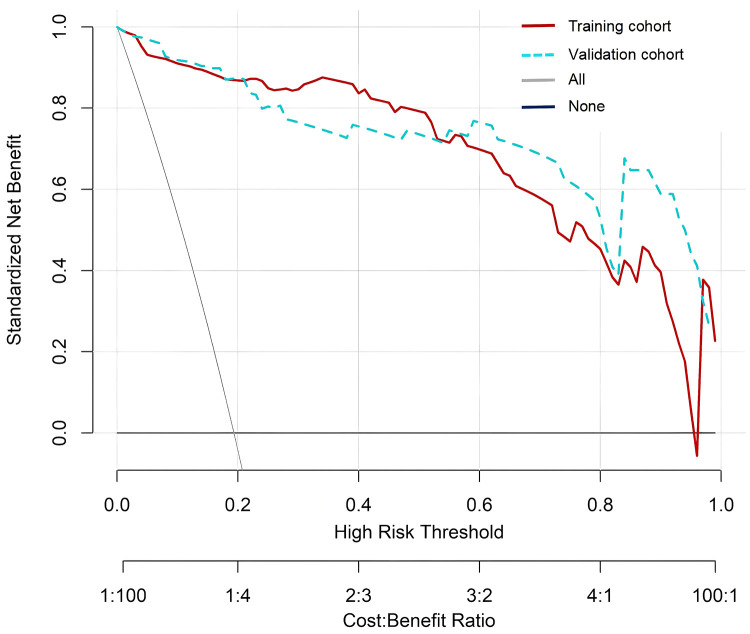
DCA of nomogram. DCA, decision curve analysis.

In the Hosmer Lemeshow test, the training cohort had a *χ*^2^ = 7.717, *P* = 0.462, and the validation cohort had a *χ*^2^ = 8.391, *P* = 0.396. This result indicates that the predicted results are close to the observed results. The ROC curve in the training cohort showed good discriminability (AUC: 0.985; 95% CI: 0.973–0.997); The discriminative performance of nomogram was validated in the validation cohort (0.975; 0.955–0.996) (Figure 4). In addition, calibration curve analysis shows that there is good consistency between the predicted probability and the observed occurrence of NPE in both the training cohort and validation cohort (Figure 5). DCA demonstrated the clinical practicality of the model (Figure 6).

## Discussion

The results of this study showed that the risk of NPE in the training cohort and validation cohort after HICH was 19.3% (53/274) and 22.4% (34/152), respectively. This is consistent with the previously reported incidence rate (2%∼42.9%) (6–9). Meanwhile, we established and validated an effective and useful nomogram for predicting NPE in high-risk patients with HICH. This new prediction tool has been successfully validated in different cohorts and demonstrated good discriminative and calibration capabilities. This model includes six risk factors, including cerebral hemorrhage volume, heart rate, GCS score, PaO2/FiO2, LAC, and BNP levels.

We found that the higher the amount of cerebral hemorrhage, the higher the risk of developing NPE. We believe that this may be due to excessive bleeding causing an increase in intracranial pressure in patients, stimulating sympathetic nervous system excitation and releasing vasoactive substances such as adrenaline and endothelin into the systemic circulation. This leads to increased pulmonary vascular filtration pressure and endothelial cell damage in patients, resulting in increased pulmonary capillary permeability, which in turn causes NPE and exacerbations (8–10, 12). Chacón-Aponte et al. (12) also showed that higher levels of cerebral hemorrhage can easily compress peripheral nerves, flush into the ventricles, and cause lung diseases such as NPE and respiratory distress through complex brain lung pathways.

It is currently recognized that the occurrence of NPE is related to the mediation of the sympathetic nervous system (13). When the sympathetic nervous system is excited, the ratio of alpha receptors to beta receptors in the lungs is imbalanced, causing damage to the cell membrane through a series of pathological and physiological changes (13, 14). This also leads to rapid sympathetic discharge, increased systemic blood pressure, and bradycardia induced by pressure reflex, as well as enhanced venous return; resulting in pulmonary vascular congestion characterized by interstitial edema, accumulation of exudate within the alveoli, and alveolar hemorrhage (13–15). This study found that increased heart rate (OR: 1.067; 95% CI: 1.005∼1.133) is an independent risk factor for cerebral hemorrhage combined with NPE. It is speculated that increased heart rate in patients with cerebral hemorrhage complicated with NPE is related to sympathetic nervous system excitation, and can therefore be used as a predictive factor for cerebral hemorrhage combined with NPE (15–17). This is consistent with the research results of Chen et al. (16) and Nastasovic et al. (17)

The GCS score is a core indicator for assessing the degree of consciousness impairment (18). The lower the GCS score in nomogram, the higher the patient's score, indicating that the lower the GCS score, the higher the risk of developing NPE. The GCS score can indicate the severity of HICH in patients, with lower scores indicating neurological disorders, blood flow disorders, etc., which can lead to pulmonary neurological dysfunction, hemodynamic changes, and ultimately NPE (18, 19). Jawaid et al. (19) found that patients with GCS scores ≤ 8 were significantly associated with pulmonary edema. Meanwhile, relevant evidence also suggests that studies have shown that low GCS scores reflect brainstem damage that directly disrupts sympathetic nervous system regulation pathways, leading to rapid progression of pulmonary edema (18–20).

PaO2/FiO2 ≤ 200 mmHg is one of the diagnostic criteria for NPE (6, 7). Our results also indicate that a decrease in PaO2/FiO2 is one of the independent risk factors for NPE. This is mainly due to the strong stress response in the patient's body after brain injury, increased sympathetic nervous system excitability, resulting in increased respiratory rate and decreased PaO2; Moreover, increasing the FiO2 did not significantly increase PaO2, resulting in a decrease in PaO2/FiO2 (21). Therefore, for HICH patients with gradually decreasing PaO2/FiO2, even if there are no relevant clinical symptoms, attention should be paid to the occurrence of NPE (21, 22). Therefore, we suggest that when the respiratory rate of HICH patients shows a progressive increase and PaO2/FiO2 shows a progressive decrease, the occurrence of NPE should be highly suspected even if there are no typical clinical manifestations of NPE, after excluding primary diseases of the heart, lungs, and kidneys and without aspiration or excessive or rapid infusion (21–23). Meanwhile, our results indicate that the higher the LAC level, the higher the risk of NPE. The study by Satoh et al. (24) also showed that elevated serum lactate levels within one hour after subarachnoid hemorrhage are an independent factor associated with early onset of NPE. We believe that this is because the stress response after cerebral hemorrhage causes sympathetic nervous system excitation, leading to increased respiratory rate, shortened pulmonary arteriovenous oxygen exchange time, decreased PaO2/FiO2, increased anaerobic metabolism in the body, and increased LAC (24, 25).

BNP is a peptide synthesized and secreted by atrial myocytes (26). The main function of BNP is to dilate blood vessels, thereby regulating the water salt balance in the body (26–28). There are reports that BNP levels are significantly increased in patients with cerebral hemorrhage combined with NPE, but there is no abnormality in cardiac function. Therefore, it is suspected that the increase in BNP levels may be related to the formation of NPE (27, 28). This study also showed that patients with concurrent NPE had significantly elevated BNP, which is an independent risk factor for HICH combined with NPE (OR: 1.026; 95% CI: 1.009–1.044), but patients did not have any heart dysfunction. The elevation of BNP reflects the overall disorder of the “nerve-heart-lung” axis after central nervous system injury (28). So, in HICH patients, we also recommend carefully monitoring changes in cardiac biomarkers (such as BNP) until they are confirmed to have returned to near normal values.

Although BNP is commonly associated with cardiac dysfunction, BNP elevation can also occur after severe acute brain injury due to neuro–cardiac interactions and catecholamine-mediated myocardial stress (12, 15, 29). Therefore, BNP in our model should be interpreted as a marker of cardiopulmonary stress in the context of hypertensive intracerebral hemorrhage rather than evidence of cardiogenic pulmonary edema (27, 29). In this study, the diagnosis of NPE was based on a composite of clinical manifestations, imaging features with typical evolution, and systematic exclusion of cardiogenic and alternative pulmonary causes, rather than BNP alone (7, 22, 29).

Hematoma location may be pathophysiologically relevant to NPE, particularly when hemorrhage involves regions closely related to autonomic regulation, which could amplify sympathetic activation and cardiopulmonary stress (5, 15, 29). In our cohort, location was recorded and evaluated as a candidate predictor; however, it was not retained after LASSO selection, suggesting limited incremental predictive value beyond hematoma volume and neurological severity. In addition, the proportion of brain stem hemorrhage was relatively small and location was classified using broad anatomical categories, which may reduce statistical power to detect an independent association. Future multicenter prospective studies with larger sample sizes and more granular imaging phenotyping are warranted to further elucidate the independent role of hematoma location in NPE risk.

Although the respiratory manifestations of NPE may be severe, it can rapidly progress to respiratory failure and cardiovascular failure (29). However, appropriate intensive care and supportive treatment typically lead to significant improvement within 48–72 h (6–10, 15, 17, 29). However, due to the unclear pathogenesis of NPE, there is a lack of intervention measures targeting the pathogenesis of NPE. For example, although mechanical ventilation can improve blood oxygen levels, it cannot block pulmonary vascular endothelial damage caused by sympathetic nervous system disorders; Moreover, high PEEP (>15 cm H₂O) may increase the risk of barotrauma. Although drug therapy (such as furosemide) can alleviate pulmonary edema, it may lead to insufficient blood volume. Although hematoma removal surgery can clear cerebral hematomas, the surgery itself cannot directly reverse the pathological process of NPE (5, 20, 23). In addition, NPE has strong clinical heterogeneity, and some patients may experience multiple organ failure due to “overtreatment” (such as excessive use of vasoactive drugs); Delaying treatment may miss the golden intervention window (23, 29). Early identification of high-risk individuals for NPE is crucial for the prognosis of patients (5, 10, 29).

The nomogram can assist healthcare professionals in early diagnosis of related diseases (30). To our knowledge, this is the first nomogram developed for NPE after HICH. The six predictive factors it contains were established in a well characterized HICH patient training cohort. We validated the developed Nomogram model using various validation methods such as C-index, calibration curve, ROC curve, and decision curve, which enhances its credibility. The validation results showed that the C-indices of the training and testing cohorts were 0.980 (95% CI: 0.966–0.994) and 0.976 (95% CI: 0.952–1.000), respectively. The calibration curves of the two cohorts are closer to the diagonal (ideal curve); The AUC values under the ROC curve were 0.985 (95% CI: 0.973–0.997) and 0.975 (0.955–0.996), respectively. The decision curve shows a high net benefit value, further proving that the nomogram has good prediction accuracy for predicting NPE.

This study has several limitations. First, this was a single-center retrospective study; although a temporally separated validation cohort was established, both cohorts were derived from the same institution. Therefore, the current validation should be interpreted as single-center internal temporal validation rather than external validation, and institution-specific factors may have influenced model performance. Multicenter prospective external validation is required before broad clinical implementation. Second, despite standardizing predictor measurement to the first assessment after admission to reduce time-dependent bias, variability inherent to retrospective documentation cannot be completely avoided. Third, we used a complete-case approach; however, exclusions due to missing baseline data were uncommon (6 in the development period and 3 in the validation period), and the potential impact is likely limited. Fourth, treatment- and intervention-related variables were not systematically available for modeling, which may introduce residual confounding and restrict causal interpretation. Finally, the nomogram showed exceptionally high discrimination; although a parsimonious LASSO-based strategy with temporal internal validation was applied, some optimism cannot be fully excluded. The high performance may partly reflect a relatively homogeneous case-mix and strong early physiological signals, and partial overlap between PaO₂/FiO₂ and the diagnostic framework of NPE may have contributed to discrimination. In addition, non-linear relationships between continuous predictors and NPE risk were not formally assessed, and outcome adjudication relied on retrospective documentation and available cardiac evaluations; therefore, misclassification cannot be completely excluded. Future larger multicenter prospective studies with standardized treatment data, more rigorous adjudication, and appropriate modeling of non-linearity are warranted to further refine and validate the model.

## Conclusion

There are many factors that can affect the occurrence of NPE after HICH, some of which are simultaneously exposed, significantly increasing the risk of NPE after HICH. This study analyzed NPE after HICH and found that cerebral hemorrhage volume, heart rate, GCS score, PaO2/FiO2, LAC, and BNP levels were independent risk factors for NPE. The Nomogram model constructed from this has certain predictive value. It helps healthcare workers quickly assess NPE risks and optimize treatment processes during the emergency phase.

## Data Availability

The original contributions presented in the study are included in the article/Supplementary Material, further inquiries can be directed to the corresponding author.
